# Towards Dynamic Contrast Specific Ultrasound Tomography

**DOI:** 10.1038/srep34458

**Published:** 2016-10-05

**Authors:** Libertario Demi, Ruud J. G. Van Sloun, Hessel Wijkstra, Massimo Mischi

**Affiliations:** 1 Biomedical Diagnostics Laboratory, Signal Processing Systems group, Faculty of Electrical Engineering, Eindhoven University of Technology, Eindhoven the Netherlands; 2 Academic Medical Center Amsterdam, Amsterdam, the Netherlands

## Abstract

We report on the first study demonstrating the ability of a recently-developed, contrast-enhanced, ultrasound imaging method, referred to as cumulative phase delay imaging (CPDI), to image and quantify ultrasound contrast agent (UCA) kinetics. Unlike standard ultrasound tomography, which exploits changes in speed of sound and attenuation, CPDI is based on a marker specific to UCAs, thus enabling dynamic contrast-specific ultrasound tomography (DCS-UST). For breast imaging, DCS-UST will lead to a more practical, faster, and less operator-dependent imaging procedure compared to standard echo-contrast, while preserving accurate imaging of contrast kinetics. Moreover, a linear relation between CPD values and ultrasound second-harmonic intensity was measured (coefficient of determination = 0.87). DCS-UST can find clinical applications as a diagnostic method for breast cancer localization, adding important features to multi-parametric ultrasound tomography of the breast.

Nowadays, there is growing interest in the development of imaging techniques which are capable of detecting and localizing angiogenesis and neovascularization. These processes induce specific changes in the microvascular structure, represent an established marker for tumours, and also provide indications of tumour aggressiveness^1^. In particular, dynamic contrast-enhanced ultrasound (DCE-US) imaging shows promise, with many novel approaches focusing on the direct and/or indirect characterization of the microvasculature. However, when considering the various imaging options, several challenges emerge for imaging the breast.

Typical ultrasound contrast agents (UCAs) are gas-filled microbubbles with diameters ranging between 1 and 10 μm; they are therefore suitable for intravenous injection and can flow through the smallest microvessels. This phenomenon is exploited by super-localization ultrasound techniques which overcome the diffraction limit and are capable of imaging the microvasculature with a spatial-resolution as small as 8–12 μm 2,3. Additionally, these techniques provide access to accurate velocity maps, thus offering a powerful tool for the study of microvascular blood flow. However, the relatively long imaging time needed (e.g., >2 minutes per plane^2^), the influence of motion, and the difficulties in imaging and localizing single microbubbles in deep tissue, pose limitations to the use of these modalities in large organs.

Another recently-developed imaging method is acoustic angiography^4^. With this technique, high spatial-resolution images (in the order of 100 μm) are obtained using tenfold higher frequencies than with normal DCE-US echo-imaging. Once again, the key lies in the UCAs peculiar response to ultrasound. Because of their highly nonlinear behaviour, UCAs can backscatter high-frequency broadband echo signals (15–35 MHz), which can be used to achieve improved spatial-resolution^5^. However, frequency-dependent attenuation practically constrains the applicability of this technique to relatively small depths, such as those required for imaging the peripheral zone of the prostate (1–2 cm).

Other techniques chose a different path rather than targeting high spatial-resolution.

Standard DCE-US imaging (i.e., Harmonic Imaging, Pulse Inversion, and Amplitude Modulation) is an echo graphic technique, which in essence exploits variations in the second harmonic amplitude to generate real-time images of UCA kinetics when flowing through the vasculature^6^,^7^. In particular, the analysis of microbubble flow-dynamics through the vessels can be used to reveal changes in the vasculature itself. To this end, several techniques which are based on the quantification of parameters related to UCA perfusion and dispersion have been proposed^8^,^9^,^10^,^11^,^12^. Although the typical DCE-US spatial resolution is in the order of 1 mm, hence unsuitable for imaging microvascular changes, these techniques are still able to infer relevant information in relation to the ‘angiogenetic switch’ (the transition from a pre-vascular to a vascularized tumour phenotype) required for cancer to grow beyond 1–2 mm in diameter^13^,^14^.

However, performing a handheld DCE-US is particularly challenging for the breast, and makes imaging highly dependent on the skills of the operator. The development of dynamic contrast-specific ultrasound tomography may represent a breakthrough in breast cancer diagnostics, allowing for a more-practical, faster, and less operator-dependent imaging procedure^15^,^16^. Moreover, imaging artefacts affect standard DCE-US and limit UCA quantification and localization accuracy^17^,^18^.

In order to address these shortcomings, and thanks to a newly discovered UCA marker, a contrast-specific imaging modality named cumulative phase delay imaging (CPDI) has recently been proposed for contrast-enhanced ultrasound tomography^19^,^20^. CPDI is based on the fact that the diverse physical phenomena behind nonlinear propagation in tissue and UCA are producing a different delay accumulation between the second harmonic (2H) and fundamental (F0) component of the ultrasound field. In particular, a positive delay between 2H and F0 is a marker which is specific to UCAs as opposed to variations in harmonic amplitude (exploited for echo imaging), speed of sound, and attenuation (exploited for ultrasound tomography). CPDI has already proved to be capable of detecting and imaging UCA concentrations when working at pressure regimes (0.05 ≤ MI ≤ 0.2) and frequencies (2.5–3 MHz) of interest for clinical applications. However, although these initial studies demonstrated the feasibility of CPDI, its ability to capture UCA kinetics has never been demonstrated^20^.

As the achievable spatial resolution for ultrasound tomography is not expected to be comparable with that achievable with acoustic angiography and super-localization techniques, the ability of CPDI to image UCA kinetics is crucial to demonstrate its clinical significance.

## Results

**Figure 1:**
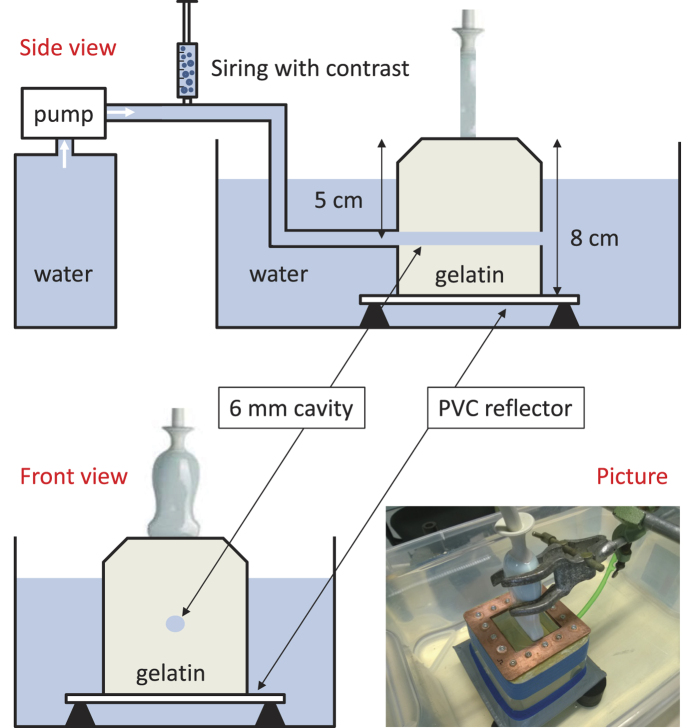
Illustrative picture, and schematics of the side and front view of the set-up are shown.

This paper reports on the first study to investigate the ability of CPDI to image UCA kinetics. To this end, the passage of repeated UCA boluses through a dedicated gelatin flow-phantom was imaged (see Fig. 1). CPDI and Harmonic Imaging (HI) were simultaneously applied to each bolus passage (in tomography and echo mode, respectively) in order to perform a comparison between the two methods and to analyze the relation between CPD values and harmonic intensity. In this paper, HI specifically refers to the pulse-echo imaging technique which relies on band-pass filters for the extraction of the second harmonic component.

Each bolus resulted from a 5-mL injection with a 240-μL/L UCA dilution. A clinically approved agent, SonoVue® (Bracco, Milan, Italy), was used for this study. To generate and store the ultrasound fields, a ULA-OP^21^ ultrasound open research platform was employed together with a LA332 linear array probe (Esaote, Firenze, Italy). Insonating frequency, mechanical index (MI), and frame rate were set at 2.5 MHz, 0.07, and 8 Hz, respectively.

### The ability of CPDI to capture UCA kinetics and qualitative comparison with HI

**Figure 2:**
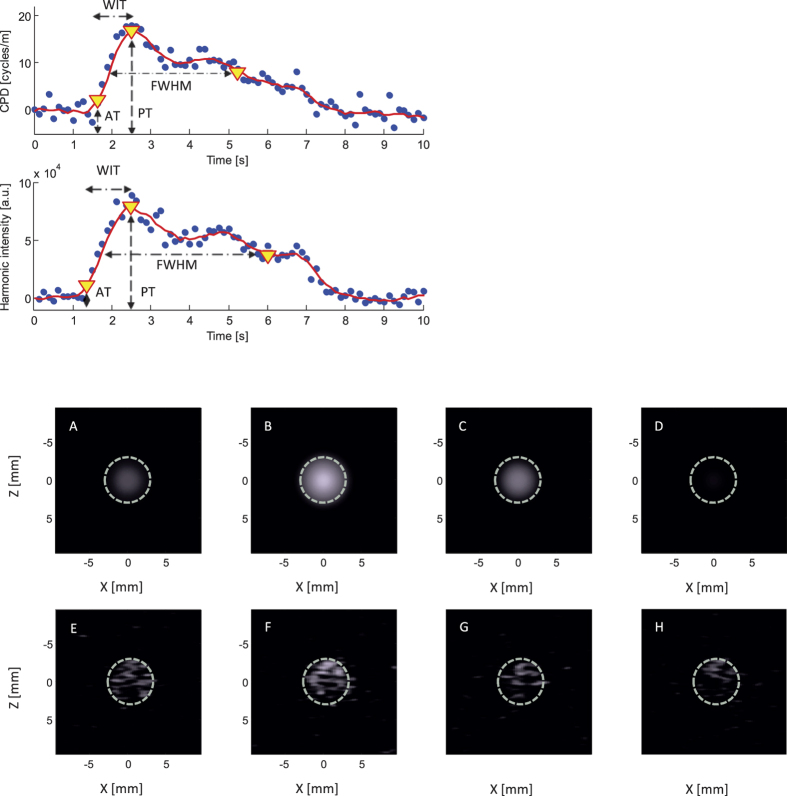
The ability of CPDI to capture UCA kinetics and qualitative comparison with HI. At the top left, time-intensity curves (TICs) obtained from cumulative phase delay (top) and harmonic imaging (bottom). Raw (blue dots) and processed (red lines) curves are shown. At the bottom, cumulative phase delay (**A–D**) and harmonic (**E–H**) images of an ultrasound contrast agent bolus passage are shown at different time instants.

Figure 2 shows, for both CPDI and HI, an example of a time intensity curve (TIC), together with the corresponding images obtained at different time instances. Processed curves (red lines) are obtained using a 0.75-s moving average filter. Various TIC features which are commonly used to quantify UCA kinetics^9^ are also marked: arrival time (AT), peak time (PT), wash-in time (WIT), and full width half maximum (FWHM). For both imaging modalities, each TIC was obtained by averaging the image values over the surface corresponding to the location of the channel cross-section (indicated by white dashed lines). Qualitatively, the two imaging methods provided similar results.

### Reported linear relation between CPD values and 2H intensity and quantitative analysis

**Figure 3:**
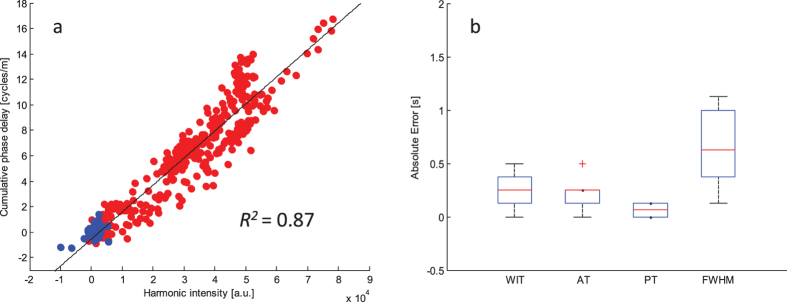
Reported linear relation between CPD values and 2H intensity and quantitative analysis. (**a**) Scatterplot of the cumulative phase delay values compared to the harmonic intensity values as obtained for the measured TICs. Blue points refer to values obtained before the arrival time. The red points refer to values measured during the passage of the bolus. (**b**) Box-plot showing the absolute error in seconds in comparison with quantitative analysis of multiple TICs features: wash-in time (WIT), arrival time (AT), peak time (PT), and full width half maximum (FWHM).

Figure 3a shows the relation between CPD values and second harmonic intensity as a scatterplot of the data-points of all TICs. The blue points refer to the values obtained before the arrival time. A linear relation is observed with a coefficient of determination equal to 0.87. Figure 3b shows a box-plot analysis of the absolute error values calculated for different TIC features when comparing CPDI and HI data. The feature that shows the highest absolute error is FWHM, with a median absolute error value equal to 0.625 s. As for the other features, the median absolute error was 0.25 s, 0.25 s, and 0.0625 s for AT, WIT, and PT, respectively.

The relative standard deviation of the area under the dilution curves, a feature which can be used for perfusion assessment, was also calculated across all the measurements and was found to be equal to 0.18 and 0.17 for CPDI and HI, respectively. Moreover, the mean and standard deviation of the Pearson’s correlation coefficient between TICs extracted by CPDI and HI were equal to 0.91 and 0.04, respectively. Overall, the quantitative analysis of TICs obtained with both imaging methods provides equivalent results.

### Comparison with speed of sound changes and attenuation due to UCA

Currently, no contrast-specific modality exists for ultrasound tomography. In fact, speed-of-sound variations and attenuation (normally used to perform ultrasound tomography^15^,^16^) due to UCAs can be confused (same range) with those caused by different tissue types^20^,^22^,^23^,^24^. Conversely, CPDI is based on a marker specific for UCAs, thus opening the way to dynamic contrast-specific ultrasound tomography.

**Figure 4:**
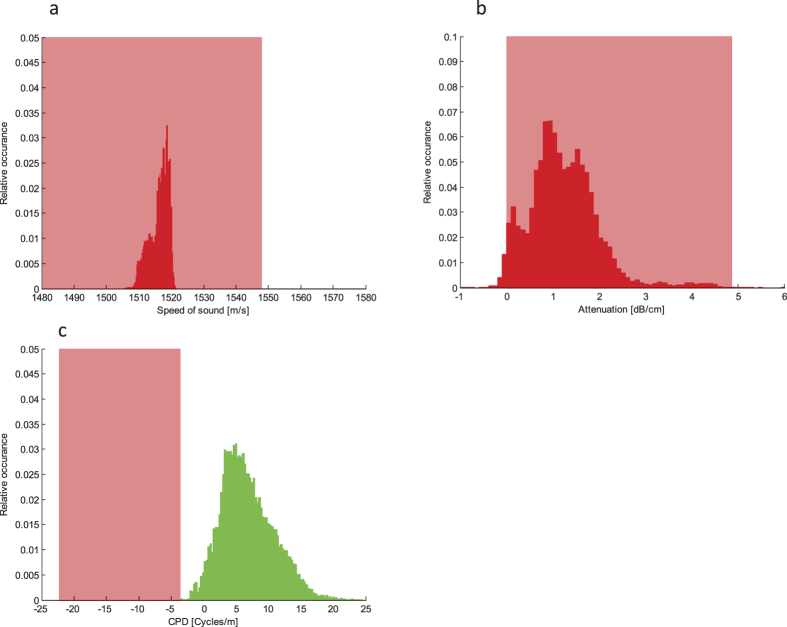
Comparison with speed of sound changes and attenuation due to UCA. Histograms of (**a**) speed of sound, (**b**) attenuation (measured at 2.5 MHz), and (**c**) CPD values (expressed in cycles per mm) as obtained from our experiment. Histograms are obtained by analyzing all the data over the area which corresponds to the location of the channel cross-section during the bolus passage. Each figure also shows the range (red box) of values representative for breast tissue for each parameter: 1480–1548 m/s for speed of sound, 0.74–4.575 dB/cm for attenuation, and −22.5 to −3.56 cycles/m for CPD.

Figure 4 shows the histograms of (a) speed of sound, (b) attenuation (measured at 2.5 MHz), and (c) CPD values (expressed in cycles per m) obtained from our experiment. These histograms were generated by analyzing all the data over the area corresponding to the location of the channel cross-section. Each figure also shows the range of values typically found in breast tissue for each parameter. The data for attenuation and speed of sound in breast tissue were obtained from^22^ and^23^, respectively. The tissue range shown in Fig. 4(c) was calculated considering blood, fat, and breast tissue, and based on frequency dispersion as derived from the models described in refs 25,26.

**Figure 5:**
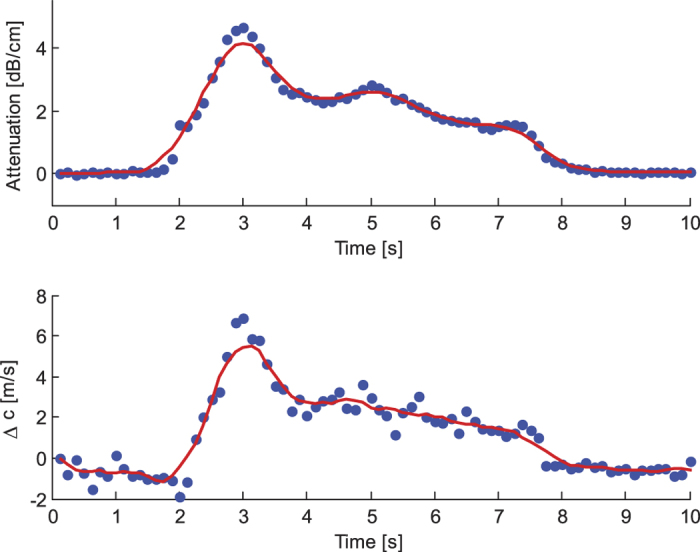
Time intensity curves displaying variations in attenuation (top) and speed of sound (bottom) with respect to baseline.

When considering speed of sound and attenuation variations, TICs can also be obtained by subtracting the baseline image, i.e., the tomographic image obtained in the absence of contrast, from all subsequent images. For illustrative purposes, Fig. 5 shows TICs obtained from speed of sound and attenuation variations corresponding to those shown in Fig. 2. Such an approach would however suffer from motion artefacts; in the presence of motion, the actual baseline image will differ from that measured before contrast enhancement.

## Discussion

In this paper the ability of CPDI to image UCA kinetics was investigated for the first time. A qualitative and quantitative comparison with HI was also performed.

Results show that CPDI can be successfully applied to image and quantify UCA kinetics. In particular, when compared to HI, equivalent results were obtained. Variations in speed of sound and attenuation due to UCA were also evaluated, and it was confirmed that they fall within tissue range.

Overall, the speed of sound and attenuation curves are very similar to the curves generated with HI and CPDI. The presence of UCA does in fact alter the speed of the ultrasound wave as well as the attenuation it experiences, the level of the second harmonic amplitude, and the time delay between the second harmonic and fundamental component. However, among all these phenomena, only the accumulation of a positive time delay between the second harmonic and fundamental component is specific to UCA.

When comparing the curves obtained with the different approaches, those based on variations in the attenuation appear to be less affected by amplitude fluctuations. However, as attenuation is not specific to contrast, and attenuation due to contrast is comparable to that exhibited by different tissue types, subtraction techniques will be required in order to exploit attenuation variations to image contrast kinetics. Such an approach is however prone to motion artefacts. Similar problems are encountered in, e.g., diffusion Magnetic Resonance Imaging^31^, especially with multi-shot acquisition, where several strategies have been developed for motion compensation. However, these strategies add an additional computational layer to the image formation process and are not error free.

CPD values measured during the passage of the UCA boluses were confirmed to be positive. This allows full tissue separation, since CPD values in tissue are inherently negative. The fact that a positive CPD value represents a marker specific to UCA is of particular importance. In principle HI could also be implemented on tomography systems which are capable of reflection tomography. However, this will not avoid typical artefacts common to HI^17^,^18^, which limit UCA quantification and localization accuracy by HI.

Unlike standard DCE-US in echo-mode, CPDI does not require any particular multi-pulse scheme. In fact, the information required for imaging is contained in the time delay between 2H and F0, which can be extracted from each single pulse^19^,^20^. Moreover, with a tomographic approach, only one-way time of flight constrains the pulse repetition frequency. Overall, these aspects allow for a higher time resolution, or faster acquisition time. Furthermore, as opposed to standard uncoded pulse-echo imaging, the pulse-length does not limit the axial-resolution. This allows for the use of longer pulses to enhance penetration and the signal to noise ratio. Moreover, the implementation of CPDI could benefit from existing speed-of-sound reconstruction algorithms which have already been developed for volumetric breast ultrasound scanners^27^,^28^; the time-of-flight could simply be replaced with CPD variations. These results are encouraging, and open the way to the development of dynamic contrast-specific ultrasound tomography, which could add important features to the multi-parametric ultrasound tomography of the breast, and improve breast cancer detection.

This paper reports on *in-vitro* results obtained by imaging the passage of UCA boluses through a cylindrical cavity surrounded by a homogeneous medium. In addition, the symmetry of the target was exploited for the tomographic reconstruction, i.e., CPD projections were assumed to be independent on the imaging angle. In real applications these two conditions do not apply and consequently impact on the image quality.

For this reason our future work, thanks to the promising results obtained, will focus on taking new measurements with a breast ultrasound computed tomography scanner on heterogeneous and more complex flow-phantoms, with the ultimate aim of transferring our technology to patients.

## Methods

### Flow phantom

A dedicated flow phantom was used to perform the experiment. A tissue-mimicking gelatin phantom (as reported in ref. 20) containing a cylindrical cavity with a 6-mm diameter was employed. A solenoid pump E410 (CEME) was used to generate the flow. A calibrated flow value equal to 0.26 L/s was employed. A fixed volume (5 mL) of SonoVue® contrast agent with a concentration equal to 240 μL/L was repeatedly injected (manually) throughout the cavity. The cavity lay at a depth of 5 cm from the location of the probe, and perpendicular to the imaging plane. Further down, at 8 cm, a PVC plate was positioned. The echoes which backscattered from the plate could therefore be recorded by the probe and used to form a tomographic image. At the same time, it was possible to measure the backscattered echoes from the microbubbles and use them to form harmonic images in echo graphic mode. This approach allowed us to compare the two imaging techniques, CPDI and HI, when (simultaneously) imaging the same bolus passage.

### Data collection

An active sub-aperture of 64 elements was used to transmit and receive the ultrasound fields, and linearly shifted over the 192-elements linear array to form a 128-line data set. The field of view was 20 mm wide and 90 mm deep. No focusing was applied in transmission and dynamic receive beamforming was used.

The post beam formed data were stored and used for the analysis. A 10-cycle pulse with its centre frequency at 2.5 MHz, and whose amplitude was modulated by a Hamming envelope, was used as a driving signal. In receive mode, a sampling frequency of 50 MHz was used. A total of 80 frames were consecutively acquired for each bolus, at a frame rate of 8 Hz. A mechanical index (MI) equal to 0.07, which was measured with a hydrophone (HGL-0400 Onda, Sunnyvale, CA) at a depth of 5 cm, was used to minimize bubble disruption. The hydrophone, with a bandwidth ranging from 250 kHz to 20 MHz, was connected to a preamplifier (AH-2010-025 Onda, Sunnyvale, CA) whose bandwidth ranged from 50 kHz to 25 MHz, which in turn was connected to a 100 MHz A/D converter (PCI-5406 National Instruments, Austin, TX). Dedicated Labview® software was implemented and used for data acquisition. The MI was calculated as the ratio of the peak negative pressure in MPa and the square root of the frequency in MHz.

### Data analysis

All data processing and analysis was performed using Matlab® R2014a.

### Harmonic Imaging

The second harmonic (2H) component was extracted by band-pass filtering. A −12 dB bandwidth around 5 MHz was selected. Subsequently, a two-dimensional spatial Gaussian filter (spatial standard deviation *σ*_x_ = 0.5 mm and *σ*_z_ = 0.25 mm) was applied to enhance the signal-to-noise ratio. To reconstruct the harmonic images, an average speed of sound equal to 1510 m/s was assumed.

### Cumulative Phase Delay Imaging

In order to measure the cumulative phase delay (CPD) between F0 and 2H, firstly, the pressure fields which had backscattered from the plate were selected by time-windowing (window length equal to 5 μs). Secondly, the two components, i.e., F0 and 2H, were extracted from the data (a −12 dB bandwidth was selected around 2.5 MHz and 5 MHz, respectively) and the corresponding envelopes were obtained by using the Hilbert transform. Thirdly, the time delay between the maxima of the fundamental and second harmonic envelopes was obtained for each line, providing a measure of CPD as a function of the lateral direction, i.e., a projection. For this experiment, the symmetry of the target was exploited for the reconstruction, in other words, CPD projections were assumed to be independent on the imaging angle. Finally, the filtered back-projection (filter type: Shepp-Logan) algorithm^29^ was used to generate 2D-CPD images.

### Time Intensity Curve

To obtain the time intensity curves from the HI and CPDI data, the image intensity values were averaged over the region corresponding to the location of the cavity. Following that, a moving average filter (window size equal to 0.75 s) was applied, and the baseline removed from the CPDI and HI TICs. The baseline was calculated as the mean value over the first 0.75 s. The average CPDI baseline was equal to −0.6 cycles/m.

### Quantitative analysis

The peak time (PT) was calculated as the time when the maximum of a TIC was observed. The arrival time (AT) was estimated as the first time the TIC amplitude exceeded 5% of its value at PT. The wash-in time (WIT) was calculated as the time between AT and PT. The full width half maximum (FWHM) was calculated as the time interval over which the TIC amplitude stayed above 50% of its value at PT. The interval corresponding to the bolus passage is defined as the time between AT and the time showing a drop in TIC-amplitude below 5% of the value at PT. The area under the curve was derived as the sum of the TIC-amplitude values over the entire time window (10 s long) multiplied by the length of the sampling interval (0.125 s).

### Speed-of-sound and attenuation estimation

By using the same filtered back-projection algorithm that was applied to perform CPDI, tomographic images based on speed-of-sound and attenuation were obtained from the variations in the time-of-flight and amplitude of the peak of the fundamental envelope, respectively. Subsequently, histograms were obtained by analysing the image values over the region corresponding to the location of the cavity, during the passage of the boluses.

### Dispersion evaluation

Values of CPD in tissue are determined by the combined effect of tissue nonlinearity and frequency dispersion^20^. Both phenomena result in a negative CPD value. However, the contribution due to tissue nonlinearity can be assumed to be negligible as compared to that due to frequency dispersion, especially for highly absorbing tissues. In fact, in line with the Kramers-Kronig relationship^30^, dispersion and absorption are interlinked. In conclusion, to evaluate CPD values, absorption measures reported in ref. 26 for blood, fat, and breast tissue were used in combination with the theoretical model in ref. 25.

## Additional Information

**How to cite this article**: Demi, L. *et al*. Towards Dynamic Contrast Specific Ultrasound Tomography. *Sci. Rep.* **6**, 34458; doi: 10.1038/srep34458 (2016).
